# Supplementary material for: Barriers to and enablers of childhood immunization uptake in Ethiopia’s Amhara, Oromia, and Somali Regions: A multi-perspective qualitative study
Source: PLOS Glob Public Health. 2026 Jun 5;6(6):e0006554. doi: 10.1371/journal.pgph.0006554 (PMC13240881; doi:10.1371/journal.pgph.0006554)
Supplement: S1 Text — This file contains the data collection instruments. (DOCX) [file pgph.0006554.s001.docx]

**S1 Appendix**

**Data collection tools: FGD, IDI and KII Guides**

# **FOCUS GROUP DISCUSSION GUIDE**

Date: ___________  Time: ___________

Place where the discussion was held (e.g., kebele office, school compound, health center, etc.): ____________________________

FGD participants group (women/men?): ____________________

Number of group members: ____________

Name of Facilitator: ____________________ Name of Note taker: ____________________

District: ____________________ Name of the village: ____________________ Urban/Rural?: ____________________

| Participant’s code | Background information of the discussants | | |  |
| --- | --- | --- | --- | --- |
|  | Age | Religion | Education level |  |
| P1 |  |  |  |  |
| P2 |  |  |  |  |
| P3 |  |  |  |  |
| P4 |  |  |  |  |
| P5 |  |  |  |  |
| P6 |  |  |  |  |
| P7 |  |  |  |  |
| P8 |  |  |  |  |
| P9 |  |  |  |  |
| P10 |  |  |  |  |

**Introduction**

- Narrative welcoming participants.
- Let the participants introduce each other.
- Describe reasons for the discussion.
- Ask participants for their consent to be voice recorded for the discussion.
- Set the general ground rules for the session.

**Ground rules**

- 60 minutes (Audio recorded – interviewer and note taker)
- Speak clearly / one at a time
- Conversation / all participate
- No right / wrong answers
- Assurance of anonymity and confidentiality
  - Assurance of anonymity and confidentiality

1. **General questions**

- How familiar are you with vaccination services available in your community?

**Probe:** Please share your personal experiences with vaccination for children in your family or community.

- In your opinion, what is the importance of vaccination for children's health?
  - If the FGD participants mentioned importance of vaccination, probe for sources of information.
  - How often do they hear information about vaccinations in their community?
    - **Probe:** sources of information, places for information, barriers to information, sources of messages, types of messages.
- Do you think the community's attitude towards vaccination has changed over the years?
  - If yes, how? What makes these changes happen?
  - If no, why not?
- What should/can be done more to sustain these changes? Or bring changes in attitude of the community towards vaccination?

**2. Barriers to vaccination**

- Why are zero-dose and under-immunized children not being vaccinated?
- What factors, in your opinion, influence a parent or caregiver's decision to vaccinate their child?

**2.1. Demand-side barriers (i.e., socio-cultural, political and economic factors)**

- Are there any common beliefs or misconceptions about vaccines that you know or hear in your community?
  - If yes, tell us the beliefs and misconceptions that prevent parents to vaccinate their children.
- What role, if any, do traditional practices or cultural beliefs play in vaccination decisions within the community?
  - **Probe:** existing cultural norms? lack of decision-making power by the mother? lack of money for transport? oppositions from influential people? grandmothers?
- Do those unvaccinated and under-vaccinated children belong to any specific/special sub-populations? If so, which?
  - **Probe:** for differences by urban, remote rural, and fragile settings or by geography/community/socio-economic group.
- What should be done to change these challenges?

**2.2. Supply-side (health services) barriers**

- What challenges or barriers do you think families face when it comes to accessing and utilizing vaccination services?
- Are services available where and when needed? If not, what needs to be done?
- Can you share any personal stories or experiences related to challenges in accessing vaccination services?
  - **Probe:** availability of vaccination services in the area, vaccine availability, waiting time, distance from vaccination site, convenience of vaccination days and times, trust on immunization or vaccinator, beliefs related to vaccination, fear of side effects, any other.
- How can these barriers be addressed or what should be done to improve vaccination rates in the community?
- Role of healthcare providers:
  - How do you perceive the role of healthcare providers in promoting vaccination within your community?
  - In your opinion, what strategies can healthcare providers use to effectively address concerns about vaccines among caregivers?
- How can service delivery approaches be tailored to sustainably reach zero-dose children and missed communities in specific contexts (e.g., urban settings, conflict, remote, rural)?
- How does the community respond or support families with unvaccinated or under-vaccinated children?

**3. Enablers of vaccination**

- What are some of the positive things that promote or encourage caregivers for vaccination in this community?
  - **Probe:** the demand-related enablers (e.g., thinking and feeling; motivation; social processes – social and gender norms; and other practical issues) that encourage immunization in their community.
- What role, if any, do traditional practices or cultural beliefs play in vaccination decisions within the community?
  - **Probe:**
    - What are the traditional practices, culture, or community structures that support vaccination services use in this community?
    - How can vaccination programs be more culturally sensitive/acceptable to address these beliefs?
- How do you think community members, particularly men, can be more actively involved in promoting vaccination and ensuring that all children are vaccinated on schedule?
  - **Probe:**
    - How are men in your community commonly involved in vaccinating children in the household?
      - **Probe:** in what ways – by providing money? taking the child to vaccination sites? any other?
    - What are the roles of different members in your households in children’s vaccination?
      - **Probe:** role of mother, father, mother-in-law, other family members, younger siblings.
      - Whose approval is very important to you regarding immunizing your child? Why?
    - What are the community-led initiatives or programs or structures that you believe could improve vaccination rates?
      - (Like defaulter tracing, mobilizing the community, providing information to caregivers.)
      - Do these structures exist in this community?

**4. Intervention Ideas**

- What should be done by caregivers to improve vaccination uptake (what should be improved for caregivers)?
- What should be improved by the health services to reach under-immunized and zero-dose children?
  - **Probe:** what suggestions do you have for healthcare providers and community leaders to engage with families on vaccination?
- Is there any more anyone has to add?

**CONCEPT TESTING DISCUSSION**

In the final part of the FGD discussion, we would like to hear your opinions and thoughts on three ideas to get more children vaccinated in your community.
Please be honest about whether you think they are good or bad ideas and why.

**Question 1: Community Group Activities**

- What are your thoughts on community members coming together to identify and solve problems with getting children vaccinated?
- Who do you think should be part of these community groups, and why?

**Question 2: Engagement of Community Leaders**

- How do you feel about training community leaders to promote child vaccination and become vaccine champions?
- Who do you think would be the most influential leaders in your community on the issue of child vaccination, and why?

**Question 3: Mother-to-Mother Support Groups / Father Champions**

- How do you feel about the idea of creating separate groups for mothers and fathers to learn about child health and vaccination, and to support each other on getting their children vaccinated?
- Who do you think should lead and facilitate these groups, and why?

**Question 4: Other Interventions**

- Please tell us other ideas that you think will help children get vaccinated in your community.
  - **Probe “why” for any ideas suggested.**

**Conclusion**

Thank you for your time and valuable input. Your perspectives are quite informative and insightful in understanding and addressing vaccination disparities in the country.
I encourage you to continue being advocates for vaccination in your community.
Your feedback and responses will be used to inform strategies to enhance vaccination services for the benefit of all children in the community.

**IN-DEPTH INTERVIEW**

**Thank you for agreeing to discuss with me today.**
There are no right or wrong answers, and we are hopeful that you will be free with your opinions and feedback.

**Identification Particulars**

- **Participant’s age:** ______________________
- **Religion:** ______________________
- **Occupation:** ______________________
- **Marital status (Married / Unmarried / Divorced):** ______________________
- **Education level:** ______________________
- **Residence / Address:**
  - Region: ______________________
  - Zone: ______________________
  - Woreda: ______________________
  - Kebele: ______________________

**Introduction**

- Please tell me a little bit about yourself and your family.
  - **Probe:** How long have you been a mother? Number of children, their age, and gender? Do you work in or outside the home?
  - **Probe:** What are some of the responsibilities and challenges you face in caring for your children?

**1. General**

- What do you know about children’s vaccinations and the vaccines recommended for children in Ethiopia?
  - **Probe:**
    - Can you name any specific vaccines that are part of the routine vaccination schedule for children that are provided in the nearby health facility?
    - In your opinion, what are the importance of each vaccine you know for children's health?
    - How do you compare the prevalence of vaccine-preventable diseases now and previously?
    - How do you relate this to the introduction and expansion of vaccination?
  - If the participant mentions the names and importance of vaccines, ask:
    - How did you learn about the importance of vaccination?
    - **Probe:** Sources of information, places for information, and how often they hear about vaccinations in the community.
    - What type of information did you receive from these sources?
  - If the response is *never* or *rarely hear any information*:
    - Ask: “So, you rarely or never hear anything. Does anyone ever talk about vaccinations? Who? When? Who hears these messages?”
- What do others in your community think about vaccines?
- **Probe:**
  - As a member of the community, through your interactions in different social occasions, how do people think about child vaccination, its importance, and their interest in achieving proper vaccinations?
  - Have you personally witnessed or heard any adverse effects related to childhood vaccination in the community? What was your experience in such an occasion?
  - How do you weigh the perceived benefits of vaccination against any concerns or fears — for yourself and for the community as a whole?
- Do you think the community's awareness, attitude, and utilization of child vaccination has changed over the years?
- If **yes**, how? What makes these changes happen?
- If **no**, why not?
- If there is change in awareness:
- What should or can be done more to sustain these changes or bring further positive change in attitudes toward vaccination?

**2. Barriers to vaccination**

- What are some of the reasons for not vaccinating children that you have heard in your community?
  - **Probe:** Why are zero-dose and under-immunized children not being vaccinated?
  - What factors, in your opinion, influence a parent or caregiver's decision to vaccinate their child?
  - Each factor and the way it influences vaccination should be discussed in detail.

**2.1 Demand-side barriers** (Socio-cultural, political, and economic factors)

- **Probe:**
  - Are there any common beliefs or misconceptions about vaccines that you know or hear in your community?
    - If yes, tell us the beliefs and misconceptions that prevent parents from vaccinating their children.
  - Are there any specific barriers or circumstances that have made it difficult for you to access vaccination services?
    - **Probe for:**
      - Lack of awareness
      - Cultural norms
      - Lack of decision-making power by the mother
      - Lack of money for transport
      - Opposition from influential people or grandmothers
      - Lack of services or vaccine stockouts
      - Long waiting time or distance
      - Inconvenient vaccination time
      - Lack of trust in immunization
      - Fear of side effects
  - Do those unvaccinated and under-vaccinated children belong to any specific or special sub-populations?
    - If so, which ones and why?
    - **Probe:** Differences by urban, remote rural, or fragile settings, or by geography, community, or socio-economic group.
  - What should be done to change these challenges?
- **If the caregiver did not vaccinate her child or is a defaulter/under-immunized:**
- Are you willing to vaccinate your child in the future?
- **Probe:** Reasons for willingness or unwillingness.
- In your community, who usually makes decisions about taking children for vaccination or other health services?
- Is anyone else involved?
- Does the mother have to ask for money? Who does she ask?
- **Probe:** Role of mother, father, mother-in-law, and other family members.
- What role do fathers, men, or influential people in the community play in decisions about child vaccination?
- Do you think most fathers in your community want to be involved in decisions about child vaccination? Why or why not?

**2.2 Supply-side barriers**

- Can you describe your experiences in trying to access vaccination services for your child?
- How do you feel about the vaccination services provided at the health facility in your area?
  - How is the reception by the health workers?
- Are services available where and when needed?
  - If not, what needs to be done?
  - What obstacles or challenges do you or other community members face in vaccinating children?
  - **Probe**
    - What are the main challenges or obstacles you've faced when attempting to get your child vaccinated?
      - **Probe for:** Service-related factors (inconvenient time, service availability, trust in providers, quality of services).
    - Are you satisfied or dissatisfied? Why? What should be improved?
    - What type of support or guidance did you receive from healthcare providers or community health workers regarding vaccination?
    - How can service delivery approaches be tailored to sustainably reach zero-dose and missed communities in specific contexts (e.g., urban settings, conflict areas, remote rural)?

**3. Enablers**

- What motivates you to vaccinate your child?
- What are some of the reasons you have heard for vaccinating children?
- Where did you hear these reasons?
- Do other people say more or different reasons for vaccination? Who? What do they say?
- **Probe:**
  Demand-related enablers (e.g., thinking and feeling, motivation, social processes such as social and gender norms, and other practical issues) that encourage immunization in the community.
- What role, if any, do traditional practices or cultural beliefs play in vaccination decisions within the community?
  - **Probe:**
    - What are the traditional practices, cultures, or community structures that support vaccination in this community?
    - Are there existing community structures that support vaccination? In what ways?
    - How can vaccination programs be made more culturally sensitive or acceptable to address beliefs that affect uptake?
    - Do existing government structures and health facilities adequately address community needs for child vaccination?
      - In what way?
      - Which aspects of the service need modification, and how?

**4. Intervention ideas**

- What do you think could be done to improve the vaccination rates of zero-dose and under-immunized children in your community?
- What activities and programs do you think should be implemented to improve vaccination? Who should be responsible for each?
  - **Probe:**
    - What activities would be most effective in increasing vaccination coverage?
    - How can healthcare providers or community leaders better engage with mothers to promote vaccination?
- Is there any additional information or insights you would like to share about your experiences with childhood vaccination or being a mother of a zero-dose child?
  - **Probe:** What message or advice would you give to other mothers with zero-dose or under-vaccinated children?

**Closing**

Thank you for your time and valuable input.
Your perspectives are quite informative in understanding and addressing vaccination disparities in the community.

**KEY INFORMANT INTERVIEW GUIDE**

**INSTRUCTIONS:** The guide is interviews with for religious leaders, traditional healers, community leaders (influential persons in the community) and local health workers who have different impacts on vaccination service utilisation. The interviewer should modify the way of asking questions for specific stakeholder and their role in vaccination. The hints in the guide will notify you of some of the points that you should consider.

**Thank you for agreeing to discuss with me today on an important aspect of community health: the vaccination of children.**
I hope we will have a productive session and generate important ideas that will improve vaccination service utilization in the future.
There are no right or wrong answers, and I am hopeful that you will be free with your opinions and feedback.

**1. Background information**

Can you provide some background information about yourself and your role as a stakeholder in your community and the responsibilities you hold?
*(You can modify the role to community leader, local health worker, or other based on your interviewee’s role.)*

**Probe:**

- - How long have you been serving in this role, and what motivated you to become a religious leader or traditional healer?
  - Can you describe the key aspects of your role, especially concerning healthcare, vaccination, and community health?

**2. Role in healthcare and vaccination**

How does your role intersect with healthcare, traditional healing practices, and vaccination in your community?

- - Does your role relate to community health service utilization, especially child vaccination?
    - If yes, in what way? Could you explain your experience in detail?

**Probe:**

- - Are there specific healthcare or healing rituals, beliefs, or practices that are integral to your role?
  - How do you interact with healthcare providers, local authorities, and community members in matters related to healthcare and vaccination?
  - Can you share examples of how your religious or traditional beliefs impact healthcare decisions, especially child vaccination service utilization within the community?

**3. Current status of vaccination programs**

What is the current status of vaccination programs within your community, and how do you perceive the importance of vaccinations from a religious or traditional perspective?
*(Ask this question to local health workers to get information about the impact of religious views and traditional practices on child vaccination.)*

**Probe:**

- - Are there specific vaccination coverage rates or goals that you are aware of or aim to achieve within your community?
  - How do you communicate your views on vaccinations and their compatibility with religious or traditional beliefs to community members?
  - Can you describe any challenges or successes related to vaccination programs within your community?

**4. Influence on community healthcare decisions**

How do you influence or guide community members, especially those seeking traditional healing, regarding healthcare decisions, including vaccinations?

**Probe:**

- - What do you think are the possible opportunities or enablers to improve child vaccination in your community?
  - Are there specific messages or advice on child vaccination that you provide to individuals who approach you for healthcare guidance?
  - How do you approach discussions about vaccinations with community members who may have traditional healing preferences?
    *(This probe is for local health workers and community leaders.)*
  - Can you share examples of situations where you have played a role in shaping healthcare decisions for community members, especially regarding child vaccination?

**5. Challenges or barriers**

What are the main challenges or barriers faced by your community regarding vaccination programs and healthcare access, particularly in the context of religious or traditional beliefs?

**Probe:**

- - Are there instances where traditional healing practices have conflicted with or posed challenges to vaccination efforts?
  - Do you think the healthcare delivery system is sufficient to achieve better vaccination coverage or to reduce un-immunized and under-immunized children?
    - If yes, how?
    - If no, why not and how can it be improved?
  - How do you address vaccine hesitancy or concerns related to vaccinations within your community?
  - Have you encountered resistance or opposition to vaccination programs, and how do you navigate such situations?

**6. Strategies and solutions**

What strategies or solutions do you believe would be effective in bridging the gap between traditional beliefs and modern healthcare, including vaccinations?

**Probe:**

- - Are there opportunities for collaboration between religious or traditional healers and healthcare providers to improve healthcare access and vaccination rates?
  - How can healthcare services be more sensitive to community culture to address community-specific beliefs and practices?
  - Can you share your vision for a healthier community where traditional healing and modern healthcare coexist harmoniously?
  - Could you explain your motivation to promote and facilitate child vaccination using your role and position in the community to achieve maximum effort in improving vaccination coverage and reducing under-immunization and zero-dose vaccination?

**7. Additional insights**

Is there any additional information or insights you would like to share about your role as a religious leader or traditional healer, and the role of traditional beliefs in healthcare and vaccination within your community?

**Probe:**

- - What message or advice would you give to other religious leaders, traditional healers, or community members who are navigating the intersection of traditional beliefs and modern healthcare?

**Closing**

Thank you for your time and valuable input.
Your perspectives are informative and insightful in understanding and addressing vaccination disparities in the country.
Please feel free to add anything I may have missed from our discussion.
