# Supplementary material for: Barriers to and enablers of childhood immunization uptake in Ethiopia’s Amhara, Oromia, and Somali Regions: A multi-perspective qualitative study
Source: PLOS Glob Public Health. 2026 Jun 5;6(6):e0006554. doi: 10.1371/journal.pgph.0006554 (PMC13240881; doi:10.1371/journal.pgph.0006554)
Supplement: S2 Text — This file contains the completed COREQ checklist for this study. (DOCX) [file pgph.0006554.s002.docx]

# **S2 Appendix**

**Consolidated criteria for reporting qualitative studies (COREQ): 32-item checklist**

Framework from: Tong A, Sainsbury P, Craig J. Consolidated criteria for reporting qualitative research (COREQ): a 32-item checklist for interviews and focus groups. *International Journal for Quality in Health Care*. 2007. Volume 19, Number 6: pp. 349 – 357

| **No. Item** | **Guide questions/description** | **Response** |
| --- | --- | --- |
| **Domain 1: Research team and reﬂexivity** | | |
| *Personal Characteristics* |  |  |
| 1. Interviewer/facilitator | Which author/s conducted the interview or focus group? | The interviews were conducted by Yohannes Hailemichael, Amare Zewdie, Minahil Tadesse, Mengistu Ayenew, Tamrat Endebu, and trained data collectors, with continuous support from the Armauer Hansen Research Institute, Addis Ababa University, and the London School of Hygiene and Tropical Medicine. |
| 2. Credentials | What were the researcher’s credentials? E.g. PhD, MD | The interview team consisted of a variety of professionals, including post-doctoral research fellows, PhD students, master’s and first-degree holders |
| 3. Occupation | What was their occupation at the time of the study? | Research institute staff, University lecturers and researchers. |
| 4. Gender | Was the researcher male or female? | Male and female. |
| 5. Experience and training | What experience or training did the researcher have? | Interviewers received training in qualitative research methods and analysis through an academic educational program. They were also trained on qualitative research methods and data collection techniques with the support of the RESONATE project and CI(co-investigators) of this study. |
| *Relationship with participants* |  |  |
| 6. Relationship established | Was a relationship established prior to study commencement? | Before the start of data collection, key community influential leaders were engaged within the respective communities selected for the research. During the forum, discussions focused on the purpose of the study, what is expected from local leaders in facilitating the community entry, the role of the community as providers of evidence and the implications of the study outcome. At the health facility level, key management members were oriented about the research, expectations and implications. |
| 7. Participant knowledge of the interviewer | What did the participants know about the researcher? e.g. personal goals, reasons for doing the research | General research aims and objectives were clearly explained, the risks and benefits of participation, the purpose of voice recording, as well as the role of the interviewer and note taker during the interview. |
| 8. Interviewer characteristics | What characteristics were reported about the interviewer/facilitator? e.g. Bias, assumptions, reasons and interests in the research topic | The research team that conducted the interviews and FGDs are academic staff from research and educational institutions, with support from overseas collaborators. None of the field team members originally came from the study district but were all native speakers of the main local language in their respective regions (Amharic, Afan Oromo and Somali).  Interviewers and FGD moderators spoke Amharic, Somali, Oromo and English languages, and all interviews were conducted in the local language of the respondents (Amharic , Afan Oromo and Somali).  Interviewers consisted of people from a variety of disciplinary backgrounds, including social science, health economics and public health researchers, who have prior experience of working on immunization. |
| **Domain 2: study design** | | |
| *Theoretical framework* |  |  |
| 9. Methodological orientation and Theory | What methodological orientation was stated to underpin the study? e.g. grounded theory, discourse analysis, ethnography, phenomenology, content analysis | The conceptual framework and codebook were developed to guide the thematic analysis conducted. |
| *Participant selection* |  |  |
| 10. Sampling | How were participants selected? e.g., purposive, convenience, consecutive, snowball | In each selected district, three villages/kebeles (one urban and two rural kebeles from which the study facilities are located) were purposively selected, and IDIs, KIIs, and FGDs were conducted. The participants were selected purposively to get detailed information and were approached through health extension workers and village leaders. |
| 11. Method of approach | How were participants approached? e.g. face-to-face, telephone, mail, email | All participants were approached face-to-face. |
| 12. Sample size | How many participants were in the study? | 18 FGDs were conducted among three different social groups, including mothers(caregivers) (8 FGDs), men community members (8 FGDs) and young mothers (Ages 15 -17 years) (2 FGDs). Each FGD consisted of 6-10 participants.  A total of 23 in-depth interviews were conducted with mothers of children with different immunization status including mothers of zero dose (6 IDIs), under-immunized (5 IDIs), fully immunized (6 IDIs), and on immunization schedule (6 IDIs).  In total, 42 KIIs were conducted with different stakeholders at different levels, including community leaders at village level (7 KIIs), district level officials /politicians at district level/ (7 KIIs), Women  leaders/Youth influencers/ (8 KII), Religious leaders/traditional healers/ (8 KII), Community-based oganizations’ staff working with IDPs/ethnic groups/ people with disability representatives (5 KII), and Health workers working on immunization (7 KII). |
| 13. Non-participation | How many people refused to participate or dropped out? Reasons? | None |
| *Setting* |  |  |
| 14. Setting of data collection | Where was the data collected? e.g. home, clinic, workplace | The interviews (IDIs, KIIS) were conducted in the participants’ own homes, at health posts, health centres and their usual workplace to ensure participants felt at ease. Focus group discussions were conducted in health facilities and communal spaces, such as Kebele compounds, at health posts, and health centres. |
| 15. Presence of non-participants | Was anyone else present besides the participants and researchers? | No one was present during the FGD and interview sessions. |
| 16. Description of sample | What are the important characteristics of the sample? e.g. demographic data, date | Both genders (M and F), people with and without formal education and an age range of 15 to 68 years participated in the interviews and FGDs. Additionally, health workers who are involved in immunization at health facilities have participated. The data collection period spanned from March to April 2024. |
| *Data collection* |  |  |
| 17. Interview guide | Were questions, prompts, guides provided by the authors? Was it pilot tested? | Qualitative interview topic guides were developed for IDIs and FGDs. All guides were translated from English to local languages. The interview guides were pretested, and modification was made accordingly. |
| 18. Repeat interviews | Were repeat interviews carried out? If yes, how many? | No. |
| 19. Audio/visual recording | Did the research use audio or visual recording to collect the data? | Interviews and FGDs were audio recorded with participant consent and transcribed into English for analysis. |
| 20. Field notes | Were ﬁeld notes made during and/or after the interview or focus group? | Yes. Notes were made during interviews. FGDs were facilitated by two researchers, one of whom guided the discussion while the other documented field notes. |
| 21. Duration | What was the duration of the interviews or focus group? | IDIs, FGDs and KIIs typically lasted 30-60 minutes. |
| 22. Data saturation | Was data saturation discussed? | Yes. The redundancy of information across the themes was used to declare data saturation. |
| 23. Transcripts returned | Were transcripts returned to participants for comment and/or correction? | No |
| **Domain 3: analysis and ﬁndings** | | |
| *Data analysis* |  |  |
| 24. Number of data coders | How many data coders coded the data? | Two data coders coded the data. Two other authors reviewed the codebook and coded data. |
| 25. Description of the coding tree | Did authors provide a description of the coding tree? | Yes, the code book was developed to describe the coding elements. |
| 26. Derivation of themes | Were themes identiﬁed in advance or derived from the data? | Since the researcher used an inductive coding approach, codes were derived from the data (Themes were mainly identified in line with the aim and objectives of the study, then respective codes were schematized according to the concept) |
| 27. Software | What software, if applicable, was used to manage the data? | MAXQDA plus 2020 |
| 28. Participant checking | Did participants provide feedback on the ﬁndings? | Key findings were presented at workshops held at the national level, in the presence of program managers and immunization stakeholders. |
| *Reporting* |  |  |
| 29. Quotations presented | Were participant quotations presented to illustrate the themes/ﬁndings? Was each quotation identiﬁed? e.g. participant number | Yes, by making it anonymous |
| 30. Data and ﬁndings consistent | Was there consistency between the data presented and the ﬁndings? | Yes. |
| 31. Clarity of major themes | Were major themes clearly presented in the ﬁndings? | Yes. |
| 32. Clarity of minor themes | Is there a description of diverse cases or discussion of minor themes? | The discussion focuses on the key findings, particularly on barriers and enablers of immunization and some experiences of participants about consequences of immunization. |
