# Supplementary material for: Barriers to and enablers of childhood immunization uptake in Ethiopia’s Amhara, Oromia, and Somali Regions: A multi-perspective qualitative study
Source: PLOS Glob Public Health. 2026 Jun 5;6(6):e0006554. doi: 10.1371/journal.pgph.0006554 (PMC13240881; doi:10.1371/journal.pgph.0006554)
Supplement: Attachment — Submitted filename: Review for PLOS Global Health.docx [file pgph.0006554.s004.docx]

**Review**

This is a well-written paper about a pertinent issue in Ethiopia. I recommend the following to enhance clarity.

**Introduction**

Line 64: What efforts to address under immunization have been made already? Any evidence of political commitment?

Good comparison between the under-vaccinated in Ethiopia and Africa to highlight the problem.

Give a brief description of the immunisation system in Ethiopia, including the vaccine schedules. What are development armies, and what role do they play in the vaccination system?

No reference is provided for the large-scale intervention programme mentioned here. Has it been implemented already?

**Study setting**

Why were eight districts chosen? Who selected them as priority districts and why?

Was the study conducted to explore the most appropriate design for an already planned large-scale intervention to improve equitable coverage? Please state it clearly.

Line 171: Did interviewees choose where they wanted to be interviewed?

What criteria were used, and how were the FGD participants selected? From vaccination registers? From clinics? From the community? And by whom?

How were KII participants selected?

How was confidentiality maintained?

**Discussion**

Can the following sentences be divided into two for better clarity?

Sentence starting from Line 572 to 575

Sentence starting from Line 615 to 619.
