# Supplementary material for: Barriers to and enablers of childhood immunization uptake in Ethiopia’s Amhara, Oromia, and Somali Regions: A multi-perspective qualitative study
Source: PLOS Glob Public Health. 2026 Jun 5;6(6):e0006554. doi: 10.1371/journal.pgph.0006554 (PMC13240881; doi:10.1371/journal.pgph.0006554)
Supplement: Attachment — Submitted filename: Response to Reviewers.docx [file pgph.0006554.s005.docx]

**Response to Reviewers**

Dear Editors,

Thank you for the opportunity to revise our manuscript titled ‘Barriers to and enablers of childhood immunization uptake in Ethiopia's Amhara, Oromia, and Somali Regions: A multi-perspective qualitative study’ [PGPH-D-25-03042] for potential publication in the PLOS Global Public Health journal. We are grateful to the reviewer for their comments, which have allowed us to spend more time on it. In response to specific points, we provided a marked copy with tracked changes. In addition, we have detailed our responses under specific reviewer requests below. We trust that we have answered the comments sufficiently and look forward to your decision.

Best regards,

Study authors

**Journal Requirements**

1. Please include a complete copy of PLOS’ questionnaire on inclusivity in global research in your revised manuscript. Our policy for research in this area aims to improve transparency in the reporting of research performed outside of researchers’ own country or community. The policy applies to researchers who have travelled to a different country to conduct research, research with Indigenous populations or their lands, and research on cultural artefacts. The questionnaire can also be requested at the journal’s discretion for any other submissions, even if these conditions are not met.  Please find more information on the policy and a link to download a blank copy of the questionnaire here: <https://journals.plos.org/globalpublichealth/s/best-practices-in-research-reporting>. Please upload a completed version of your questionnaire as Supporting Information when you resubmit your manuscript.

**Response:** We have included a complete copy of PLOS’ questionnaire on inclusivity in global research as Supporting Information in the revised manuscript.

2. Please amend your detailed Financial Disclosure statement. This is published with the article. It must therefore be completed in full sentences and contain the exact wording you wish to be published.
1. Please clarify all sources of funding (financial or material support) for your study. List the grants (with grant number) or organizations (with url) that supported your study, including funding received from your institution.
   2. State the initials, alongside each funding source, of each author to receive each grant.
   3. State what role the funders took in the study. If the funders had no role in your study, please state: “The funders had no role in study design, data collection and analysis, decision to publish, or preparation of the manuscript.

4. If any authors received a salary from any of your funders, please state which authors and which funders.

If you did not receive any funding for this study, please simply state: “The authors received no specific funding for this work.”

**Response:** We have amended the detailed Financial Disclosure statement as requested. Here it is for your convenience:

This study was funded by a grant from GSK to the Save the Children Fund (<https://www.gsk-savethechildren.com/>) [author initials: MS, PV, TA, AC, SM] , and a subgrant from Save the Children to LSHTM and AHRI (grant number 104369) [author initials: AZ, MTB, MA, TE, MD, AA, MA, CRM, NA, YH]. GSK had no role in study design, data collection and analysis, decision to publish, or preparation of the manuscript.

3. In the online submission form, you indicated that Excerpts of the qualitative data supporting the findings of this study are available from the corresponding author upon reasonable request. However, full transcripts cannot be shared due to confidentiality agreements.

All PLOS journals now require all data underlying the findings described in their manuscript to be freely available to other researchers, either 
1. In a public repository, 
2. Within the manuscript itself, or 
3. Uploaded as supplementary information.

This policy applies to all data except where public deposition would breach compliance with the protocol approved by your research ethics board. If your data cannot be made publicly available for ethical or legal reasons (e.g., public availability would compromise patient privacy), please explain your reasons by return email and your exemption request will be escalated to the editor for approval. Your exemption request will be handled independently and will not hold up the peer review process, but will need to be resolved should your manuscript be accepted for publication. One of the Editorial team will then be in touch if there are any issues.

**Response:** Thank you for your suggestion. We have sent a return email requesting an exemption (22^nd^ January 2026). Here is the text of email requesting an exemption:

Dear PLOS Global Public Health Editorial Team,

We would like to request an exemption from sharing the full transcripts of the data underlying the findings in a public repository or as supplementary information, on the grounds that this would breach confidentiality agreements with study participants. In the participant information sheet and consent form, which the study participants read and signed, consecutively, we have not been granted permission to share the research data with other researchers.

Below are the relevant parts of the participant information sheet and consent forms signed by participants, which were approved by the ethics committees:

**Participant information sheet:**

***What will happen to information collected about me?*** 
*All information collected about you will be kept private and it will not be shared with anyone else. Only a small number of researchers within the study team in Ethiopia and at the London School of Hygiene & Tropical Medicine will be allowed to look at information collected.*

**Consent form:**

- *I agree that the research team may use my data for future research and understand that any such use of identifiable data would be reviewed and approved by a research ethics committee.  (In such cases, as with this project, data would not be identifiable in any report).*

Furthermore, we would like to request an exemption from presenting the full data underlying the findings presented in the manuscript on the grounds of unfeasibility. The supporting quotations we included in the manuscript illustrate the findings presented, but do not constitute the full volume of data underlying them. This is because this qualitative study is very large, with almost 200 participants enrolled *(n=198)*, and it would not be practical to present all the data in the body of the manuscript.

Many thanks for considering our exemption request.

4. If the reviewer comments include a recommendation to cite specific previously published works, please review and evaluate these publications to determine whether they are relevant and should be cited. There is no requirement to cite these works unless the editor has indicated otherwise.

**Response:** Thank you for your comment. The reviewers did not recommend any previously published works.

**Reviewer comments**

**Review**

This is a well-written paper about a pertinent issue in Ethiopia. I recommend the following to enhance clarity.

**Response:** Thank you for your comment. We have addressed all your comments and specified where changes were made by referring to line numbers in the manuscript version with tracked changes.

**Introduction**

Line 64: What efforts to address under immunization have been made already? Any evidence of political commitment?

**Comment:** Line 64: What efforts to address underimmunization have been made already? Any evidence of political commitment?

**Response:** Thank you for your comment. We have added a paragraph (now the third paragraph, lines 64 - 77) briefly describing the efforts to address underimmunization as evidence of political commitment by the Government of Ethiopia and its partners.

**Comment:** Good comparison between the under-vaccinated in Ethiopia and Africa to highlight the problem.

**Response:** Thank you for your comment.

**Comment:** Give a brief description of the immunisation system in Ethiopia, including the vaccine schedules. What are development armies, and what role do they play in the vaccination system?

**Response:** Thank you for your suggestion. We added a brief description of the immunisation system in Ethiopia, including the vaccine schedules and some information about health development armies (lines 92 – 101).

**Comment:** No reference is provided for the large-scale intervention programme mentioned here. Has it been implemented already?

**Response:** Thank you for your comment. At the time of the study, the intervention had not yet been designed or started. However, it is currently under implementation). We have added a reference to their website.

**Comment:** Why were eight districts chosen? Who selected them as priority districts and why?

**Response:** Thank you for your comment. The regions and districts were purposively selected by the implementing agency and the Ethiopian Ministry of Health based on the number of zero-dose and under-immunized children, the occurrence of vaccine-preventable diseases such as measles, and the presence of displaced or migratory or conflict- or drought-affected populations. We added this information to the “study setting” section (lines 133 – 136)

**Comment:** Was the study conducted to explore the most appropriate design for an already planned large-scale intervention to improve equitable coverage? Please state it clearly.

**Response:** Thank you for your comment. The study was conducted study conducted to explore the most appropriate design for an already planned large-scale intervention to improve equitable coverage (lines 136 – 138)

**Comment:** Line 171: Did interviewees choose where they wanted to be interviewed?

**Response:** Thank you for your comment. The interview locations were mutually agreed upon by the interviewee and the interviewer (lines 202 – 203)

**Comment:** What criteria were used, and how were the FGD participants selected? From vaccination registers? From clinics? From the community? And by whom?

**Response:** Thank you for your comment. We made minor edits to further clarify selection in lines 169 – 174 (Study participant section). Here is the amended text for convenience.

*In each selected district, three kebeles (the smallest administrative units in Ethiopia) were purposively chosen, comprising one urban and two rural kebeles. Participants for interviews were purposively selected by the research team, guided by health extension workers and community leaders in each kebele. The selection criteria for IDI and FGD participants were their children's immunization status, as reported by their families and from the health facilities in the kebeles.* *For KIIs, we selected participants based on their roles in immunization service delivery, and their social influence within the community. The final sample size for each method and participant group was determined by data saturation.*

**Comment:** How were KII participants selected?

**Response:** Thank you for your comment. We made minor edits to further clarify selection in lines 174 - 176 (Study participant section). Here is the amended text for convenience.

*For KIIs, we selected participants based on their roles in immunization service delivery, and their social influence within the community. The final sample size for each method and participant group was determined by data saturation.*

**Comment:** How was confidentiality maintained?

**Response:** Thank you for your comment. We added more text to the “ethical considerations” section, lines 247 – 252. Here is the amended text for convenience.

*To ensure confidentiality, we excluded personal identifiers, including names, addresses, phone numbers, and facility names, from all written materials. We restricted access to raw data, audio recordings, and transcripts to the research team only, protecting them through password-secured files and secure storage systems. We also presented findings in aggregated and summary form to ensure that individual participants could not be identified*

**Comment:** Can the following sentences be divided into two for better clarity?

Sentence starting from Line 572 to 575

Sentence starting from Line 615 to 619.

**Response:** Thank you for your comment. We divided the two sentences as suggested for better clarity (now lines 610–613 and 654-659).
