# Supplementary material for: Barriers to and enablers of childhood immunization uptake in Ethiopia’s Amhara, Oromia, and Somali Regions: A multi-perspective qualitative study
Source: PLOS Glob Public Health. 2026 Jun 5;6(6):e0006554. doi: 10.1371/journal.pgph.0006554 (PMC13240881; doi:10.1371/journal.pgph.0006554)
Supplement: Attachment — Submitted filename: Response_to_Reviewers_auresp_2.docx [file pgph.0006554.s006.docx]

**Response to Reviewers**

Dear Editors,

Thank you for the opportunity to revise our manuscript titled ‘Barriers to and enablers of childhood immunization uptake in Ethiopia's Amhara, Oromia, and Somali Regions: A multi-perspective qualitative study’ [PGPH-D-25-03042] for potential publication in the PLOS Global Public Health journal. We are grateful to the reviewer for their additional comments. In response to specific points, we provided a marked copy with tracked changes. In addition, we have detailed our responses under specific reviewer requests below. We trust that we have answered the comments sufficiently and look forward to your decision.

Best regards,

Study authors

**Journal requirements**

If the reviewer comments include a recommendation to cite specific previously published works, please review and evaluate these publications to determine whether they are relevant and should be cited. There is no requirement to cite these works unless the editor has indicated otherwise. 

Please review your reference list to ensure that it is complete and correct. If you have cited papers that have been retracted, please include the rationale for doing so in the manuscript text, or remove these references and replace them with relevant current references. Any changes to the reference list should be mentioned in the rebuttal letter that accompanies your revised manuscript. If you need to cite a retracted article, indicate the article’s retracted status in the References list and also include a citation and full reference for the retraction notice.

**Response:** Thank you for this suggestion. The reviewer comments did not include a recommendation to cite specific previously published works.

In addition, we conducted a thorough review of the entire reference list to ensure completeness, accuracy, and consistency with the Vancouver referencing style. We verified journal names, citation details, and DOI formatting for all references.

We also screened all cited articles for retraction status. No retracted publications were identified among the references included in the revised manuscript. Therefore, no further action was required in this regard.

All modifications made to the reference list have been reflected in the revised manuscript through track change, and detailed here:

- We removed this reference which was erroneously added: Guidance W. Ethics and governance of artificial intelligence for health. World Health Organization. 2021:1-165.
- We added these three references to the discussion:

37. World Health Organization. Understanding the behavioural and social drivers of vaccine uptake - WHO position paper. Weekly Epidemiological Record. 2022;97(20):209–24.

38.	Jalloh MF, Patel P, Sutton R, Kulkarni S, Toure M, Wiley K, et al. Qualitative assessment of caregiver experiences when navigating childhood immunisation in urban communities in Sierra Leone. BMJ Open. 2022;12(5):e058203.

39.	Dubé E, Laberge C, Guay M, Bramadat P, Roy R, Bettinger J. Vaccine hesitancy: an overview. Hum Vaccin Immunother. 2013;9(8):1763-73.

**Reviewer 2 comments**

**Comment:** In the ethical considerations section, I recommend clarifying the age range of the youth influencers included in the study.

**Response:** Thank you for this valuable suggestion. We have revised the ethical considerations section to clarify the age range of the youth influencers included in the study. Specifically, we now state that all youth influencers were aged 18 years or older. As such, they were considered adults, and the standard ethical procedures, including informed consent, were applied. This clarification has been incorporated into the revised manuscript (lines 251 – 253 in tracked change version).

**Comment:** I have two comments on the discussion section.1) The authors write "We also found evidence that these three categories are inextricably connected and mutually affect each other." However, there is no additional discussion on how the categories are connected and mutually affect each other once they are described. I recommend adding in a short paragraph or a couple of sentences to demonstrate this point.

**Response:** Thank you for this helpful comment. We agree that the interrelationship among the three categories requires further clarification. In response, we have added a concise paragraph in the discussion section to explicitly explain how these categories are interconnected and mutually influence one another, drawing on examples from our findings and the wider literature to illustrate these dynamics (lines 599 – 610 in tracked change version)

**Comment:** 2) Regarding the first category on the influence of "knowledge, perceptions and socio-cultural norms on vaccination uptake," I recommend clarifying differences between knowledge and misconceptions. For example, do people not know what the number of recommended doses of a vaccine are, or could you be more specific about what the misconception about this is (e.g., two are recommended but you only actually need one). If there is no difference, I recommend defining what you mean by knowledge (misconceptions and sources of information?) or changing the title of the section to reflect the focus on misconceptions and sources of information specifically.

**Response:** Thank you for this insightful comment. We agree that greater clarity was needed to distinguish between knowledge and misconceptions. In response, we have revised the discussion to explicitly define “knowledge” as encompassing both accurate understanding and gaps in information, and to clarify that misconceptions arise primarily from inadequate or incomplete knowledge, as well as exposure to unreliable sources of information. We have also refined the relevant section to better articulate this relationship and included illustrative examples to improve clarity (lines 615 – 622 in tracked change version)
